# Effect of bedside comprehensive ability training on teaching and training in operating room

**DOI:** 10.3389/fmed.2025.1743984

**Published:** 2026-01-23

**Authors:** Yi Zhong, Wenjun Bu, Qifan Li, Lili Zheng

**Affiliations:** 1 Department of Hepatobiliary Surgery, Nanfang Hospital, Southern Medical University, Guangzhou, Guangdong, China; 2 Department of Anesthesia Operation Center, Nanfang Hospital, Southern Medical University, Guangzhou, Guangdong, China

**Keywords:** bedside comprehensive ability training, core competence, nursing, operating room teaching, problem-based learning

## Abstract

**Aim:**

To assess the effect of bedside comprehensive ability training on teaching and training in operating room.

**Methods:**

One hundred and thirty female clinical nurses who joined our hospital from January 2019 to December 2021 were randomly assigned to a control group and an observation group. The control group adopted problem-based learning (PBL), while the observation group adopted bedside comprehensive ability training. The training effect, core competence evaluation results, self-comprehensive ability as well as teaching satisfaction of 2 groups of nurses were compared.

**Results:**

After training, nurses in the observation group demonstrated significantly higher training scores (*p* < 0.05), higher core competence (*p* < 0.05), higher self-comprehensive ability (*p* < 0.05), higher scores of nursing ability (*p* < 0.05) and better satisfaction rate (*p* < 0.001).

**Conclusion:**

Bedside comprehensive ability training can effectively promote the core ability and training results of new clinical nurses and further improve their own comprehensive ability and nursing ability.

## 1 Introduction

With the continuous development of economy and society along with the continuous improvement of medical technology, people’s demand for health services and service quality are also constantly improving (1). The specialization of nurses has become the focus of nursing talent training (2). To ensure that nurses acquire these competencies, hospitals are working to shift from existing teaching methods that focus on lectures and rote memorization to a diverse and scientific approach. This change is conducive to improving the quality of nursing education, developing the potential of nurses, promoting all-round development, and enabling nurses to acquire professional competence (3). Therefore, there is an urgent need to find educational methods that can effectively help nurses master clinical reasoning skills.

Operating room is an important department of hospital (4). High quality perioperative nursing can not only accelerate patients’ rehabilitation, but also effectively improve the quality of surgical nursing (5). Recent reports have indicated problem-based learning (PBL) is a suitable learning approach for nurse education (6), which makes students to learn independently and describe their ideas easily, thus promoting their confidence and self-esteem (7). However, this mode of nursing practice may lead to nursing students simply imitating what their teachers demonstrate. Once they enter the clinical practice stage, they will show poor practical ability, long adaptation period and weak aseptic concept, which seriously affects the efficiency of medical work (8). The operating room involves a wide range of related nursing fields, occupies a certain nursing position, and has strong practicality. Therefore, how to better train nursing nurses is the responsibility and obligation of surgical clinical nursing.

With the reform of nursing training, the bedside comprehensive ability training method has been increasingly applied to clinical training. Some studies have defined bedside training as all training activities in front of patients, regardless of whether they are in outpatient, ward, or conference room (9). The bedside comprehensive ability training refers to the ability training of nurses to carry out bedside systematic physical examination of patients, collect data, report cases, propose nursing problems, formulate and implement nursing measures, evaluate the effect of implementation, and carry out health education (10). In the context of operating room nursing education, the concept of bedside comprehensive ability training has also been extended to include workplace-based training conducted in authentic clinical environments, even when direct patient participation is not involved. By selecting typical cases in the training process, bedside comprehensive ability training allows learners to apply previously acquired knowledge to clinical practice and facilitates the integration of theoretical learning with clinical experience (11).

From an educational perspective, bedside comprehensive ability training is grounded in experiential and situated learning theories, which emphasize learning through direct participation in real clinical contexts (12). Clinical education research has shown that learning situated in authentic practice environments enables students to integrate theoretical knowledge with real-world skills, leading to improved performance and professional readiness (13). In the context of nursing education, experiential teaching methods have been used to enhance learners’ clinical judgment, decision-making, and procedural competence by providing opportunities for direct practice and contextualized learning (14). Compared with PBL, which emphasizes the cognitive processing of information, workplace-based and experiential learning strategies place greater emphasis on the development of skills and behaviors through real or highly realistic practice experiences, making them particularly suitable for training operating room nurses whose competence depends on procedural proficiency, communication, and team-based performance (15).

This study compared the effect of traditional training and bedside comprehensive ability training in operating room nursing training and explored a more suitable model for operating room nursing training.

## 2 Materials and methods

### 2.1 General information

One hundred and thirty female clinical nurses who joined our hospital from January 2019 to December 2021 were enrolled and assigned to either the control group or the observation group (*n* = 65 per group). Participants were allocated at enrollment using a simple random assignment approach. The random sequence was generated by a member of the research team who was not involved in training delivery or outcome assessment. Participants were enrolled sequentially at the time of entry into the standardized pre-job training program, and group assignment was implemented at enrollment. Allocation concealment was not implemented.

The inclusion criteria were: (1) newly registered clinical nurse with less than 12 months of clinical experience post-graduation; (2) assigned to rotational training within the surgical departments; and (3) voluntary agreement to participate. Exclusion criteria included nurses with prior operating room working experience or those transferred from other hospitals during the study period.

A total of 130 newly recruited clinical nurses were assessed for eligibility and all met the inclusion criteria. All participants were randomized and allocated to either the control group (PBL, *n* = 65) or the observation group (bedside comprehensive ability training, *n* = 65). All participants received the allocated intervention, completed the training program, and were included in the final analysis, with no exclusions, dropouts, or loss to follow-up. Supplementary Figure 1 shows the CONSORT flow diagram of participant enrollment, randomization, and analysis. No significant difference was seen between 2 groups in terms of gender, age as well as educational background (*p* > 0.05; Table 1). All nurses signed the informed consent.

**Table 1:** General data of nurses in 2 groups.

Groups	*N*	Age (years)	Education level		
			Technical secondary school	Junior college	Undergraduate
Control group	65	23.05 ± 3.40	19	32	14
Observation group	65	23.70 ± 3.50	17	33	15
*t*/*χ*^2^		1.074		0.161	
*p*		0.284		0.922	

### 2.2 Methods

The training for both groups was conducted as part of the routine standardized pre-job clinical training program for newly recruited nurses. The intervention was implemented in a structured and continuous manner over a period of 6 weeks, with each session lasting approximately 3 h. Both groups underwent training during the same training phase and for the same overall duration, with the training method being the only difference between groups.

#### 2.2.1 Control group

PBL training method, that is, before class, the teacher helped to clarify the learning objectives and then selected cases according to the patients’ condition and learning objectives. The nurses were asked to consult relevant materials, seek answers independently, and discuss their findings with other nursing students. Finally, the teacher affirmed the correct views of nursing students, analyzed the problems and clarified the causes and results of the problems.

Observation group: bedside comprehensive ability training method, and the specific steps were as follows:

1. Teaching preparation: A complete operating room system was prepared, including hand brushing room, dressing room, operating room, instrument dressing room and other workshops. The operating room was equipped with surgical patient model, operating table, instrument table, shadowless lamp, anesthesia machine, electrocardiogram (ECG) monitor, surgical instruments, surgical sheets and other surgical equipment and items, allowing trainees to practice standardized operating room procedures in a highly realistic but simulated environment without involving real patients.
2. Operating room nurses with clinical operation experience and school surgical nursing teachers with rich teaching experience served as training teachers, unified demonstration teaching actions, formulated training teaching plans, and scientifically and reasonably arranged the teaching contents of the training courses. In actual teaching, teachers required students to strictly implement the sterile requirements of operating room and relevant rules and regulations. Operating room nurses with clinical operation experience and school surgical nursing teachers with rich teaching experience served as training instructors. All instructors received prior alignment training and followed a unified teaching plan, including standardized demonstration procedures, learning objectives, and assessment criteria, to ensure consistency across training sessions.
3. Teaching process: three teachers demonstrated how to change clothes correctly as itinerant nurses, instrument nurses and surgeons → prepare sterile tables → wash hands before operation → wear surgical clothes → wear gloves → disinfect the skin in the operation area → lay operation sheets → count surgical instruments and tidy up sterile tables → transfer instruments during operation → tidy up instrument dressings after operation → take off surgical clothes and gloves. The teacher led 12 students to do technical exercises in small groups. The purpose of this step was to make students proficient in various operations. Teachers prepared some learning videos of common operations, such as kidney surgery, subtotal gastrectomy and thyroid surgery for nursing students to learn and discuss before class. At the same time, nursing students were required to think and practice more in the training process, timely correct non-standard behaviors, repeated training, so that nursing students form standardized operating habits. After all nursing students completed the training operation, the students raised their confusion and questions about the training and made self-summary and self-reflection. In this process, teachers evaluated, explained and supplemented the content summarized by students. Finally, the nursing students were comprehensively evaluated from the aspects of operation process, aseptic concept, hands-on ability and teamwork ability. The training topics were standardized in advance through a unified teaching plan developed by experienced operating room nurses and nursing educators. Standardized teaching objectives, operating procedures, and training content were applied consistently throughout the training process. For bedside comprehensive ability training, typical surgical cases relevant to operating room nursing were selected based on predefined suitability criteria rather than disease complexity, ensuring comparability of training exposure among participants. Patient selection was based on suitability for training rather than individual disease complexity.

It should be clarified that the bedside comprehensive ability training in this study was conducted in a real operating room environment using surgical patient models and standardized procedural demonstrations. No real patients were involved in the training process. The term “bedside” in this context refers to workplace-based training conducted in an authentic clinical setting rather than direct patient-facing bedside instruction.

### 2.3 Observation indicators

All outcome assessments were conducted by instructors involved in the training process using predefined evaluation criteria. Independent assessors were not used. Due to the nature of the intervention, blinding of instructors and participants to group allocation was not feasible. To ensure consistency, the same assessment standards and procedures were applied to both groups during the same assessment period.

1. The training scores of nurses were compared, mainly including nursing writing, nursing operation training and responsible holistic nursing. The score ranged from 0 to 100 points, with the higher score representing better training of nurses.
2. The core competence of nurses was compared using the core competence questionnaire. The main contents of the questionnaire included professional construction and development ability, critical clinical thinking ability, support and interpersonal communication ability, clinical nursing ability as well as good personal characteristics. The score ranged 0–220 points, with the higher score representing better core competence of nurses.
3. The comprehensive abilities of nurses were compared. The invigilator assessed the nurses’ ability to score on improving their technical skills and consolidating their theoretical knowledge. The score range was 0–100, the higher the score, the better the comprehensive abilities of nurses.
4. An author-developed nursing ability questionnaire was used to evaluate nurses’ clinical nursing ability. The questionnaire focused on key dimensions of operating room nursing practice, including emergency rescue ability, nurse–patient communication ability, instrument operation proficiency, and mastery of nursing knowledge. The score range was 0–100, the higher the score, the stronger the nursing ability of nurses.
5. A self-made satisfaction questionnaire was adopted to investigate the satisfaction of the rotating residents with the teaching work, mainly including the enthusiasm of the teaching process and the effectiveness of the teaching on the improvement of professional skills. The full score of the questionnaire was 100. The questionnaire was distributed, filled in and recycled on site. The final score of 90 ~ 100 was very satisfying, 70–89 was basically satisfied, and less than 70 was dissatisfied. Total satisfaction rate = (very satisfied + basically satisfied)/total number of cases × 100%.

Prior to formal implementation, the developed nursing ability questionnaire and the self-made teaching satisfaction questionnaire were pilot-tested among a small group of newly recruited nurses to assess clarity and feasibility. Internal consistency reliability was evaluated, and the Cronbach’s alpha coefficients were 0.89 for the Nursing Ability Questionnaire and 0.85 for the Teaching Satisfaction Questionnaire, indicating good internal consistency for both instruments. The Nursing Ability Questionnaire consisted of 20 items with a 5-point Likert scale (1 = Strongly Disagree, 5 = Strongly Agree). Total scores were derived by summing the responses of all items. Example items included, ‘I can effectively communicate with patients’ and ‘I can handle emergency situations with confidence,’ assessing domains such as communication, emergency response, and nursing knowledge. The pilot sample size was 50 newly recruited nurses. For the Teaching Satisfaction Questionnaire, which consisted of 15 items with a 4-point Likert scale (1 = Very Dissatisfied, 4 = Very Satisfied), total scores were also derived by summing item responses. Example items included, ‘The training was useful’ and ‘The teaching materials were adequate,’ with domains such as teaching effectiveness and material quality. The pilot sample size was 45 nursing staff, and the Cronbach’s alpha for this instrument was 0.85.

All outcome measures were assessed immediately after completion of the training program to evaluate the short-term effects of the intervention. All questionnaires were administered and completed on site immediately after training as part of routine teaching evaluation, without interfering with the training process.

The same group of instructors participated in the training of both the control and observation groups and followed a unified teaching plan with standardized teaching objectives, procedures, and assessment criteria. To minimize contamination, the two groups were trained separately during the same training phase, and no joint teaching sessions, shared practice groups, or cross-group learning activities were conducted.

### 2.4 Statistical analysis

The primary outcome of this study was the total training performance score assessed after completion of the training program. Secondary outcomes included core competence, self comprehensive ability, nursing ability, and teaching satisfaction. All data were analyzed using SPSS 20.0. Continuous variables are presented as mean ± standard deviation. Between-group comparisons for continuous variables were performed using independent-samples t-tests, and categorical variables (e.g., satisfaction rates) were compared using the *χ*^2^ test (Pearson *χ*^2^; Yates continuity correction was additionally examined for 2 × 2 tables). Within-group pre–post changes were examined descriptively, and between-group differences in post-training scores and change scores (post–pre) were used to evaluate the effect of the intervention. Randomization resulted in comparable baseline values across all outcome measures, supporting the appropriateness of post-training and change-score comparisons using independent sample t-tests. Effect sizes (Cohen’s *d*) were calculated to quantify the magnitude of between-group differences. A two-sided *p* value < 0.05 was considered statistically significant.

## 3 Results

### 3.1 Comparative result of training scores between 2 groups

**Figure 1:**
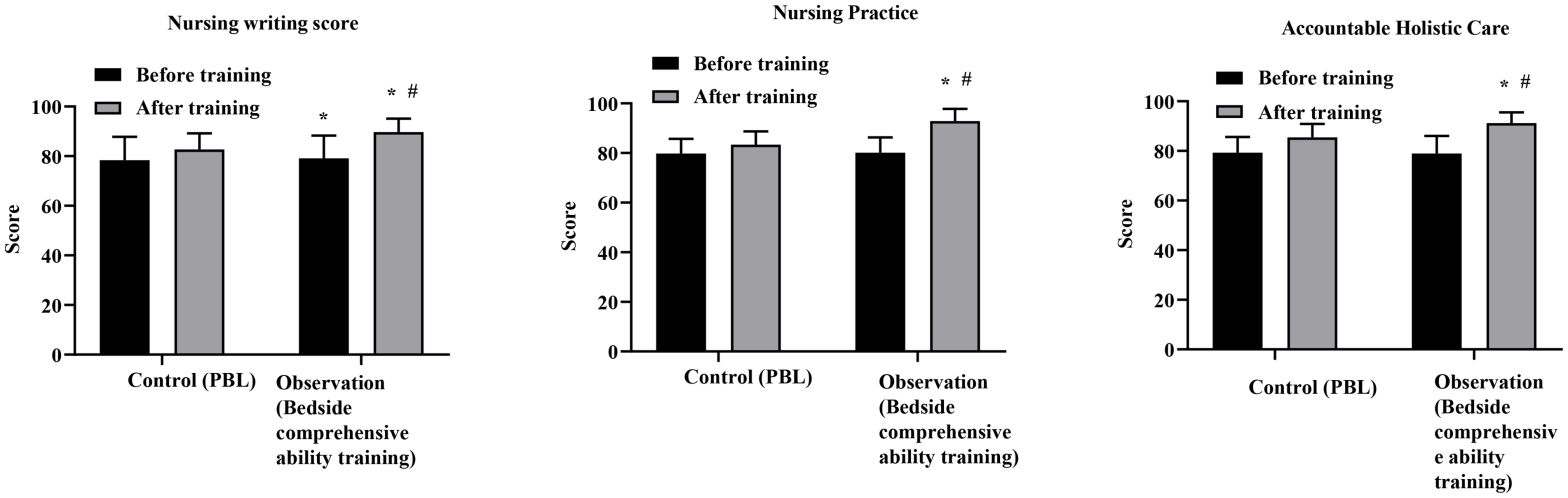
Comparison of training scores between the control group and the observation group before and after training. The control group received problem-based learning (PBL), whereas the observation group received bedside comprehensive ability training. Data are presented as mean ± standard deviation. *p < 0.05 compared with the control group at the same time point; #*p* < 0.05 compared with before training within the same group.

No significant differences were observed between the two groups in nursing writing, nursing practice, or accountable holistic care scores prior to training (all *p* > 0.05). After training, scores in all three domains were significantly higher in both groups compared with baseline. Moreover, the observation group demonstrated significantly higher post-training scores than the control group across all training score domains (all *p* < 0.05; Figure 1). Specifically, the mean post-training difference between groups was 7.0 points for nursing writing (95% CI: 4.94–9.06; Cohen’s *d* = 1.17), 9.5 points for nursing practice (95% CI: 7.75–11.25; Cohen’s *d* = 1.86), and 5.76 points for accountable holistic care (95% CI: 4.08–7.44; Cohen’s *d* = 1.18) (Supplementary Table 1). These findings indicate that bedside comprehensive ability training was associated with large and educationally meaningful improvements across multiple dimensions of training performance.

### 3.2 Comparative result of core competence between two groups of nurses

**Figure 2:**
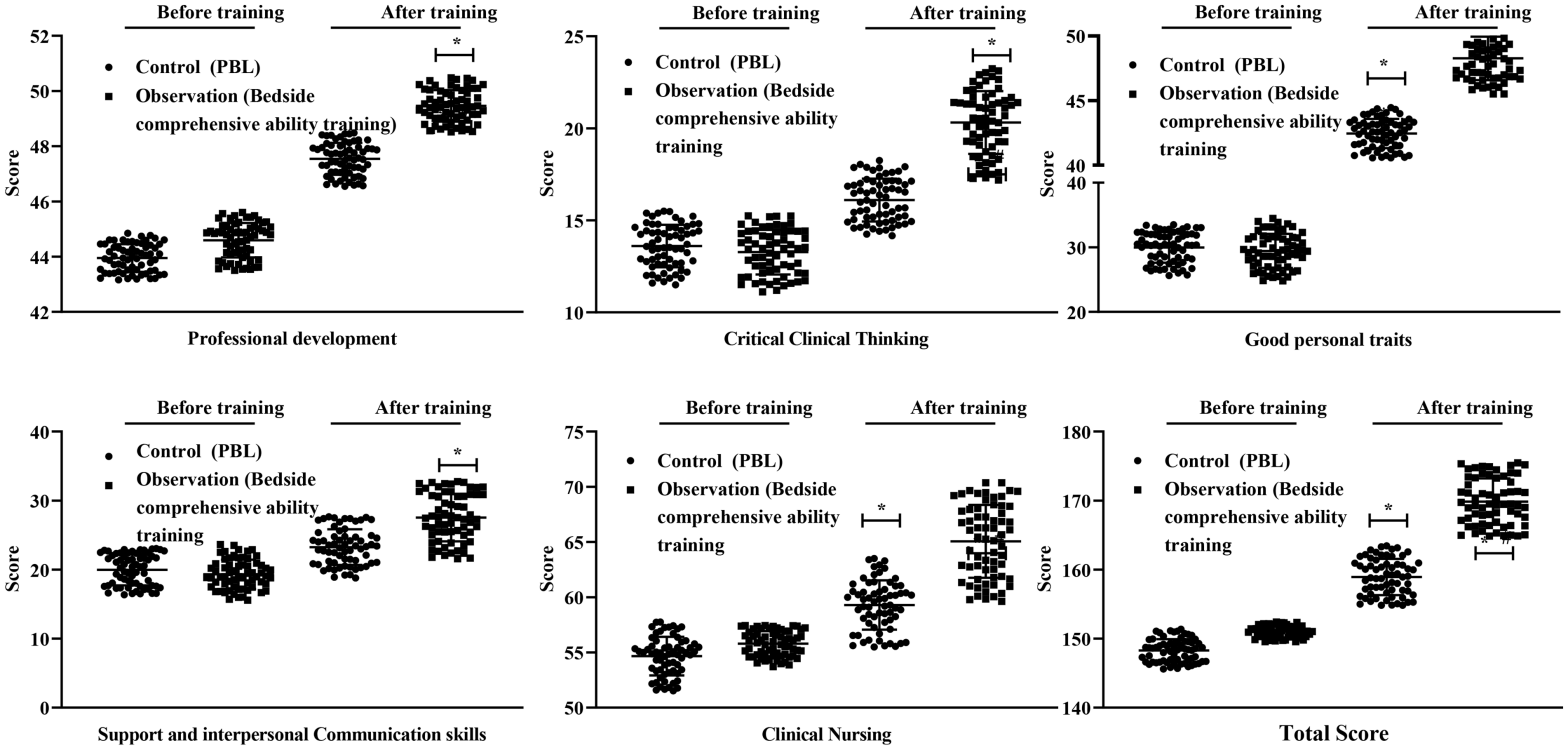
Comparison of core competence scores between the control group and the observation group before and after training. The control group received problem-based learning (PBL), whereas the observation group received bedside comprehensive ability training. Data are presented as mean ± standard deviation. *p < 0.05 compared with the control group at the same time point; #*p* < 0.05 compared with before training within the same group.

Prior to training, there were no significant differences between the two groups in any core competence dimensions, including professional construction and development ability, critical clinical thinking ability, support and interpersonal communication ability, clinical nursing ability, good personal traits, or total core competence score (all *p* > 0.05). After training, scores in all six dimensions were significantly higher in both groups compared with baseline. Moreover, the observation group demonstrated significantly higher post-training scores than the control group across all core competence domains (all *p* < 0.05, Figure 2). Specifically, the mean post-training differences between groups were 1.92 points for professional construction and development ability (95% CI: 1.73–2.12; Cohen’s *d* = 3.34), approximately 4.15 points for critical clinical thinking ability (95% CI: 3.70–4.60; Cohen’s *d* ≈ 1.60), approximately 4.37 points for support and interpersonal communication ability (95% CI: 3.58–5.16; Cohen’s *d* ≈ 1.45), approximately 6.37 points for clinical nursing ability (95% CI: 5.56–7.18; Cohen’s *d* ≈ 2.22), approximately 6.31 points for good personal traits (95% CI: 5.78–6.84; Cohen’s *d* ≈ 3.90), and approximately 11.27 points for total core competence score (95% CI: 10.22–12.32; Cohen’s *d* ≈ 3.39) (Supplementary Table 2). These results indicate that bedside comprehensive ability training was associated with consistently large to extremely large improvements across all dimensions of core competence.

### 3.3 Comparative result of self comprehensive ability between 2 groups

**Figure 3:**
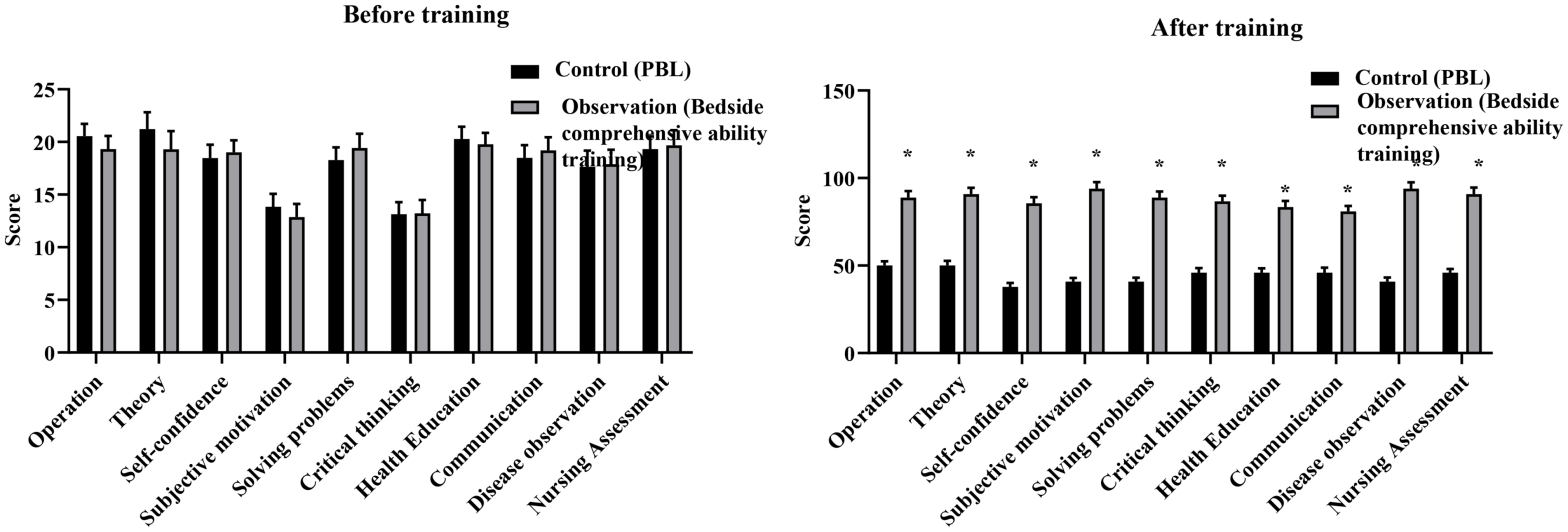
Comparison of self comprehensive ability scores between the control group and the observation group after training. The control group received problem-based learning (PBL), whereas the observation group received bedside comprehensive ability training. Data are presented as mean ± standard deviation. *p < 0.05 compared with the control group.

There was no significant difference in self comprehensive ability scores between the two groups before training (*p* > 0.05). After training, scores across all dimensions of self comprehensive ability were assessed using a detailed 0–100 point scale. The observation group demonstrated consistently and significantly higher post-training scores than the control group across all dimensions (all *p* < 0.05; Figure 3).

### 3.4 Comparative result of nursing ability between two groups of nurses

**Figure 4:**
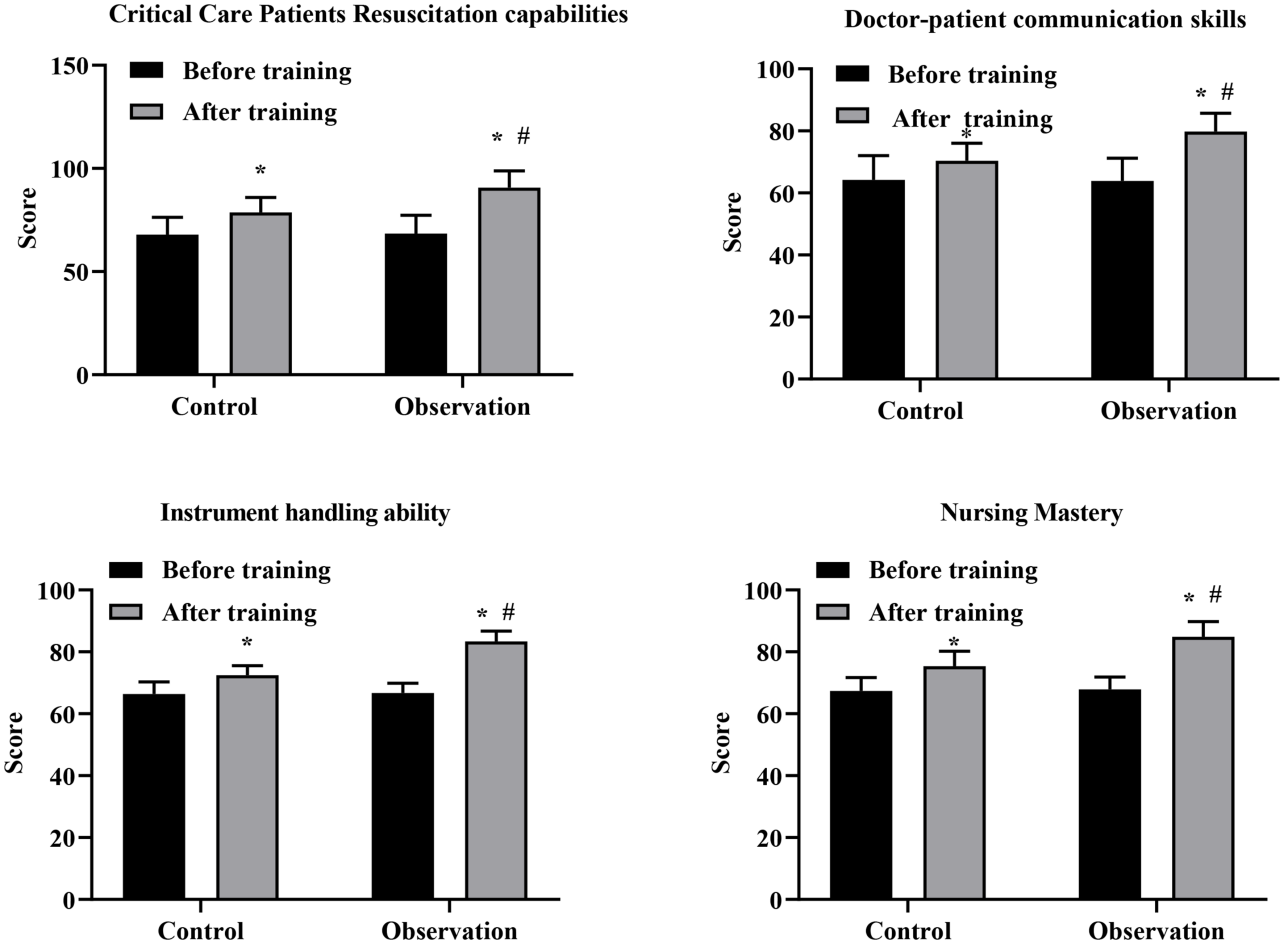
Comparison of nursing ability scores between the control group and the observation group before and after training. The control group received problem-based learning (PBL), whereas the observation group received bedside comprehensive ability training. Data are presented as mean ± standard deviation. *p < 0.05 compared with before training within the same group; #*p* < 0.05 compared with the control group at the same time point.

Prior to training, the scores of nursing ability of 2 groups were not significant (*p* > 0.05). After training, scores across all four nursing ability domains were significantly higher in both groups compared with baseline. Moreover, the observation group demonstrated consistently and significantly higher post-training scores than the control group across all nursing ability domains (all *p* < 0.05; Figure 4). Specifically, the mean post-training difference between groups was 12.02 points for critical care patients’ resuscitation capability (Cohen’s *d* = 1.56, 95% CI: 0.96–2.16), 9.40 points for nurse–patient communication ability (Cohen’s *d* = 1.63, 95% CI: 1.02–2.24), 10.95 points for instrument handling ability (Cohen’s *d* = 3.42, 95% CI: 2.58–4.26), and 9.50 points for nursing knowledge mastery (Cohen’s *d* = 1.96, 95% CI: 1.30–2.62) (Supplementary Table 3). These findings indicate that bedside comprehensive ability training was associated with large to extremely large improvements across multiple dimensions of nursing ability.

### 3.5 Comparative result of teaching satisfaction between 2 groups

There were 45 satisfied nurses and 20 dissatisfied nurses in the control group, with a satisfaction rate of 69.23%. There were 63 satisfied nurses and 2 dissatisfied nurses in the observation group, with a satisfaction rate of 96.92%. The observation group had higher teaching satisfaction than the control group (χ^2^(1) = 17.73, *p* < 0.001; Table 2).

**Table 2:** Comparison of teaching satisfaction between the two groups.

Groups	*N*	Very satisfied	Basically satisfied	Dissatisfied	Satisfaction rate (%)
Observation group	65	36	27	2	96.92
Control group	65	17	28	20	69.23
*χ*^2^ (df = 1)		17.73			
*p*		<0.001			

## 4 Discussion

This study demonstrated that, compared with the control group, the observation group achieved higher nursing writing scores, nursing operation training scores, and overall nursing responsibility system scores, indicating that bedside comprehensive ability training effectively improved the training performance of newly recruited clinical nurses. Taken together, these findings suggest that bedside comprehensive ability training enhances training performance by facilitating the integration of theoretical knowledge with real clinical practice, rather than through isolated skill rehearsal alone. Although termed “bedside comprehensive ability training,” the intervention in this study is more accurately characterized as workplace-based, simulation-supported operating room training conducted in an authentic clinical environment. Unlike traditional bedside teaching that involves direct patient interaction, this approach emphasizes experiential learning through standardized procedural demonstrations, hands-on practice with surgical models, and supervised reflection within the operating room context.

The bedside comprehensive ability training includes professional construction and development ability, critical clinical thinking, clinical nursing, support and interpersonal communication ability, medical and nursing knowledge application ability. It pays attention to whether the new nurses have keen observation and reaction ability, good psychological quality, emergency response ability and flexible use of knowledge (16). Therefore, bedside comprehensive ability training can effectively evaluate nurses’ clinical nursing ability, personal characteristics and knowledge application ability. At the same time, sound operation is also promoted during bedside training. In addition, bedside comprehensive ability training is a systematic learning and training process that requires new clinical nurses to find out case-related knowledge and accurately answer various questions raised by invigilators and patients (17). This study demonstrated that, compared with the control group, the observation group achieved higher scores in professional development ability, critical clinical thinking ability, support and interpersonal communication ability, clinical nursing ability, and personal traits. Consistently, Gu et al. suggested that the modified training program strengthened rehabilitation nurses’ core competencies (18).

Bedside comprehensive ability training can carry out planned and purposeful training for new clinical nurses, comprehensively evaluate the practical comprehensive ability of clinical nurses, and continuously deepen and consolidate their ability to combine theoretical practice with clinical practice in the process of continuous exchange, processing, analysis and search for information, so as to promote the improvement of new clinical nurses’ comprehensive ability and nursing ability (19). Consistent with previous studies (20), the observation group demonstrated significantly greater improvements across multiple dimensions of self comprehensive ability and nursing ability compared with the control group. In addition, scores for nurse–patient communication, instrument operation, and nursing knowledge mastery were significantly higher after training than before training. Importantly, the consistency of improvements across training performance, core competence, self comprehensive ability, and nursing ability suggests that bedside comprehensive ability training promotes integrated professional development rather than isolated improvements in individual skills.

This study has several limitations that should be acknowledged. First, multiple outcome variables were assessed using questionnaires, which may have increased respondent burden and potentially introduced response fatigue, thereby affecting the accuracy of participants’ self-reported data. In addition, several outcomes-particularly self comprehensive ability were self-rated, and participants were aware of their group allocation; therefore, expectation bias and response shift effects cannot be fully excluded. Second, two author-developed questionnaires were used as supplementary outcome measures. Although these questionnaires were developed based on study objectives and expert consensus, they did not undergo comprehensive psychometric validation such as construct or criterion validity testing. Therefore, findings derived from these measures should be interpreted as exploratory and hypothesis-generating, and future studies are warranted to further refine and validate standardized assessment instruments. Third, allocation concealment and assessor blinding were not implemented, and outcome assessments were conducted by instructors involved in training, which may have introduced performance and assessment bias despite the use of standardized evaluation criteria. Fourth, outcome measures were assessed immediately after completion of the training intervention, and the long-term sustainability of the observed improvements remains unclear. Future studies with extended follow-up periods are needed to examine the persistence of training effects. Fifth, this study was conducted in a single hospital and included only female nurses, which may limit the generalizability of the findings to other clinical settings or more diverse nursing populations. Moreover, several outcome measures were scored using bounded 0–100 scales, which may have introduced ceiling effects, especially for self-reported outcomes showing large post-training improvements. Therefore, the magnitude of effect size estimates for these outcomes should be interpreted cautiously, and future studies employing validated instruments with broader measurement ranges are recommended. Sixth, this study assessed multiple secondary outcomes across several domains, which may increase the risk of type I error. Although a formal multiplicity adjustment was not applied, the primary endpoint was predefined, and secondary analyses were interpreted as exploratory. The consistency and large magnitude of effects observed across related domains support the overall pattern of findings; nevertheless, these results should be confirmed in future studies using prespecified statistical models and validated outcome measures. A notable limitation concerns the interpretation of standardized effect sizes for self comprehensive ability outcomes. These measures were assessed using bounded 0–100 self-report scales, and post-training scores approached the upper scale limits for many participants. This ceiling effect substantially reduced score variability and likely inflated standardized mean differences such as Cohen’s d. Consequently, the magnitude of these effect sizes should not be interpreted as reflecting true educational effects but rather as artifacts of scale properties and response distribution. Future studies should employ validated instruments with broader measurement ranges or alternative analytical approaches, including nonparametric or baseline-adjusted models, to mitigate ceiling effects.

## 5 Conclusion

Bedside comprehensive ability training has been shown to effectively improve the core competencies and training performance of newly recruited clinical nurses. These findings underscore the value of incorporating experiential and workplace-based learning approaches in nursing education, particularly in the operating room context. Our study highlights the positive impact of this training on nurses’ practical skills, critical clinical thinking, and teamwork abilities, which are essential for effective nursing practice in high-pressure clinical environments. However, given the exploratory nature of some outcome measures and the single-center design, these results should be interpreted with caution. Further research, with multi-center studies and extended follow-up periods, is needed to validate these findings and assess the long-term impact of bedside comprehensive ability training on nursing performance.

## Data Availability

The original contributions presented in the study are included in the article/Supplementary material, further inquiries can be directed to the corresponding author.

## Supplementary material

SUPPLEMENTARY FIGURE 1 CONSORT flow diagram of participant enrollment, randomization, and analysis.
