# Supplementary figures and images for: Effect of bedside comprehensive ability training on teaching and training in operating room
Source: Front Med (Lausanne). 2026 Jan 23;12:1743984. doi: 10.3389/fmed.2025.1743984 (PMC12875977; doi:10.3389/fmed.2025.1743984)

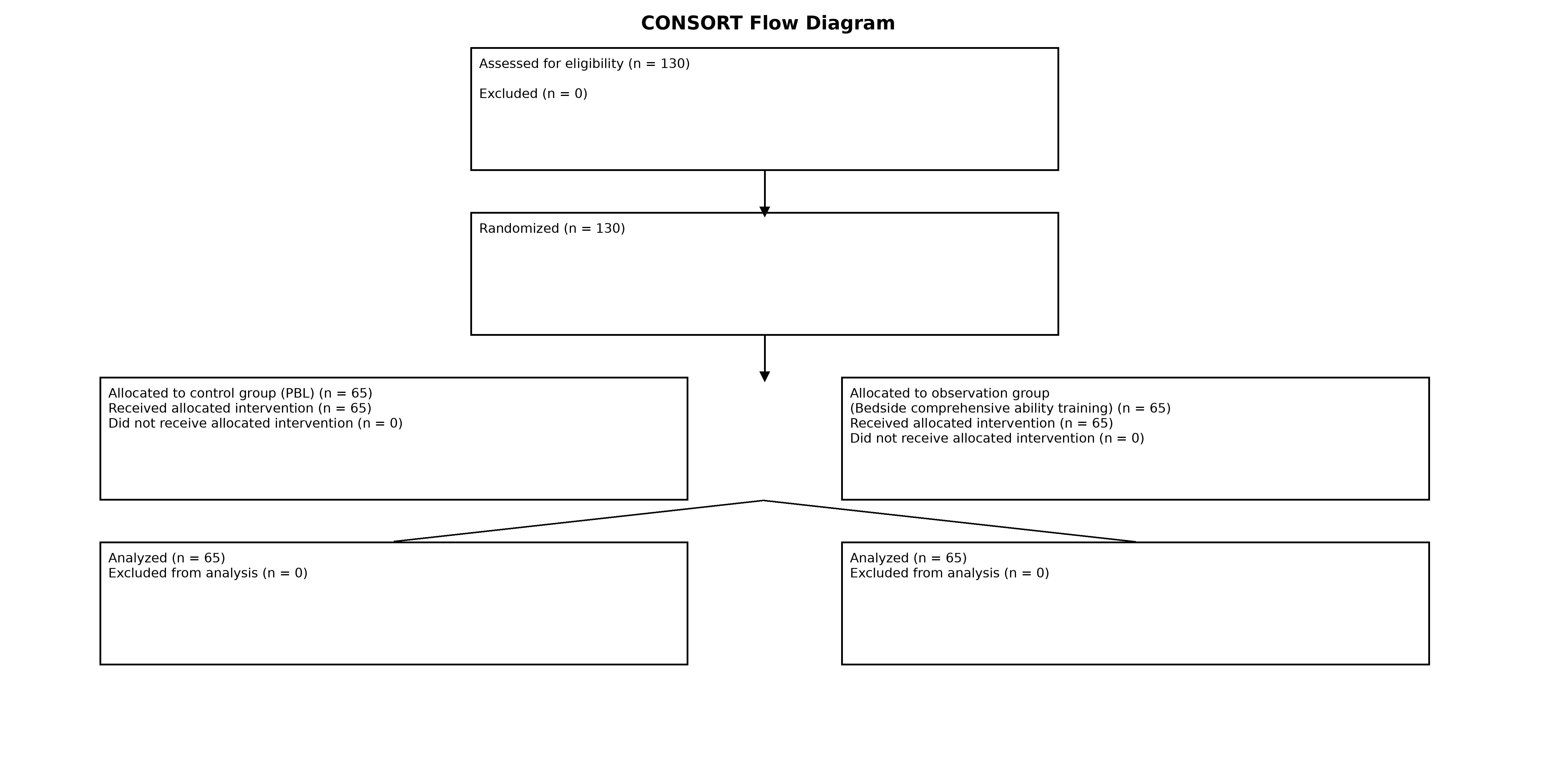

Supplement: SUPPLEMENTARY FIGURE 1 — CONSORT flow diagram of participant enrollment, randomization, and analysis. [file Image_1.tiff]
