# Supplementary material for: Effect of bedside comprehensive ability training on teaching and training in operating room
Source: Front Med (Lausanne). 2026 Jan 23;12:1743984. doi: 10.3389/fmed.2025.1743984 (PMC12875977; doi:10.3389/fmed.2025.1743984)
Supplement: Supplementary file 2 [file Table_1.DOCX]

**Supplementary Table 1 Comparison of training scores between the two groups**

| **Outcome** | **Group** | **Before Training (Mean ± SD)** | **After Training (Mean ± SD)** | **Mean Difference (After)** | **95% CI** | **Cohen’s d** |
| --- | --- | --- | --- | --- | --- | --- |
| Nursing writing | Control | 78.4 ± 9.43 | 82.7 ± 6.49 |  |  |  |
|  | Observation | 79.1 ± 9.21 | 89.7 ± 5.42 | **7.0** | **4.94-9.06** | **1.17** |
| Nursing practice | Control | 79.8 ± 5.9 | 83.4 ± 5.3 |  |  |  |
|  | Observation | 80.1 ± 6.2 | 92.9 ± 4.9 | **9.5** | **7.75-11.25** | **1.86** |
| Accountable holistic care | Control | 79.32 ± 6.27 | 85.46 ± 5.45 |  |  |  |
|  | Observation | 78.93 ± 7.11 | 91.22 ± 4.31 | **5.76** | **4.08-7.44** | **1.18** |

**Supplementary Table 2 Comparison of core competence scores between the two groups**

| **Dimension** | **Group** | **Before Training (Mean ± SD)** | **After Training (Mean ± SD)** | **Mean Difference (After)** | **95% CI** | **Cohen’s d** |
| --- | --- | --- | --- | --- | --- | --- |
| Professional construction and development | Control | 43.95 ± 0.50 | 47.54 ± 0.58 |  |  |  |
|  | Observation | 44.60 ± 0.62 | 49.46 ± 0.57 | **1.92** | **1.73-2.12** | **3.34** |
| Critical clinical thinking | Control | 13.55 ± 1.25 | 16.32 ± 1.27 |  |  |  |
|  | Observation | 13.41 ± 1.32 | 20.47 ± 1.86 | **4.15** | **3.70-4.60** | **1.60** |
| Support and interpersonal communication | Control | 19.96 ± 2.42 | 23.41 ± 2.63 |  |  |  |
|  | Observation | 19.61 ± 2.36 | 27.78 ± 3.24 | **4.37** | **3.58-5.16** | **1.45** |
| Clinical nursing ability | Control | 54.56 ± 1.78 | 59.17 ± 2.36 |  |  |  |
|  | Observation | 55.86 ± 1.46 | 65.54 ± 3.27 | **6.37** | **5.56-7.18** | **2.22** |
| Good personal traits | Control | 29.96 ± 2.32 | 42.33 ± 1.28 |  |  |  |
|  | Observation | 29.45 ± 2.61 | 48.64 ± 1.86 | **6.31** | **5.78-6.84** | **3.90** |
| Total core competence | Control | 148.27 ± 1.71 | 158.69 ± 2.80 |  |  |  |
|  | Observation | 151.05 ± 0.77 | 169.96 ± 3.79 | **11.27** | **10.22-12.32** | **3.39** |

**Supplementary Table 3 Comparison of nursing ability between the two groups**

| **Nursing Ability Domain** | **Group** | **Before Training (Mean ± SD)** | **After Training (Mean ± SD)** | **Mean Difference (After)** | **Cohen’s d** | **95% CI for d** |
| --- | --- | --- | --- | --- | --- | --- |
| **Critical care patients’ resuscitation capability** | Control | 67.90 ± 8.40 | 78.68 ± 7.30 |  |  |  |
|  | Observation | 68.40 ± 8.90 | 90.70 ± 8.10 | **12.02** | **1.56** | **0.96-2.16** |
| **Nurse-patient communication ability** | Control | 64.20 ± 7.80 | 70.40 ± 5.60 |  |  |  |
|  | Observation | 63.90 ± 7.30 | 79.80 ± 5.90 | **9.40** | **1.63** | **1.02-2.24** |
| **Instrument handling ability** | Control | 66.40 ± 3.90 | 72.44 ± 3.10 |  |  |  |
|  | Observation | 66.70 ± 3.20 | 83.39 ± 3.30 | **10.95** | **3.42** | **2.58-4.26** |
| **Nursing knowledge mastery** | Control | 67.40 ± 4.30 | 75.40 ± 4.80 |  |  |  |
|  | Observation | 67.90 ± 4.00 | 84.90 ± 4.90 | **9.50** | **1.96** | **1.30-2.62** |
